# High Mobility Group A2 protects cancer cells against telomere dysfunction

**DOI:** 10.18632/oncotarget.6938

**Published:** 2016-01-18

**Authors:** Suchitra Natarajan, Farhana Begum, Jeonga Gim, Landon Wark, Dana Henderson, James R. Davie, Sabine Hombach-Klonisch, Thomas Klonisch

**Affiliations:** ^1^ Department of Human Anatomy and Cell Science, College of Medicine, University of Manitoba, Winnipeg, Canada; ^2^ Children's Hospital Research Institute of Manitoba, Winnipeg, Canada; ^3^ Department of Biochemistry and Medical Genetics, College of Medicine, University of Manitoba, Winnipeg, Canada; ^4^ Department of Obstetrics, Gynecology and Reproductive Medicine, College of Medicine, University of Manitoba, Winnipeg, Canada; ^5^ Department of Surgery, College of Medicine, University of Manitoba, Winnipeg, Canada; ^6^ Department of Medical Microbiology and Infectious Diseases, College of Medicine, University of Manitoba, Winnipeg, Canada

**Keywords:** telomeres, HMGA2, TRF2, genomic stability, shelterin

## Abstract

The non-histone chromatin binding protein High Mobility Group AT-hook protein 2 (HMGA2) plays important roles in the repair and protection of genomic DNA in embryonic stem cells and cancer cells. Here we show that HMGA2 localizes to mammalian telomeres and enhances telomere stability in cancer cells. We present a novel interaction of HMGA2 with the key shelterin protein TRF2. We found that the linker (L1) region of HMGA2 contributes to this interaction but the ATI-L1-ATII molecular region of HMGA2 is required for strong interaction with TRF2. This interaction was independent of HMGA2 DNA-binding and did not require the TRF2 interacting partner RAP1 but involved the homodimerization and hinge regions of TRF2. HMGA2 retained TRF2 at telomeres and reduced telomere-dysfunction despite induced telomere stress. Silencing of HMGA2 resulted in (i) reduced binding of TRF2 to telomere DNA as observed by ChIP, (ii) increased telomere instability and (iii) the formation of telomere dysfunction-induced foci (TIF). This resulted in increased telomere aggregation, anaphase bridges and micronuclei. HMGA2 prevented ATM-dependent pTRF2^T188^ phosphorylation and attenuated signaling via the telomere specific ATM-CHK2-CDC25C DNA damage signaling axis. In summary, our data demonstrate a unique and novel role of HMGA2 in telomere protection and promoting telomere stability in cancer cells. This identifies HMGA2 as a new therapeutic target for the destabilization of telomeres in HMGA2^+^ cancer cells.

## INTRODUCTION

The nuclear non-histone DNA-binding protein High Mobility Group AT-hook protein 2 (HMGA2) is expressed in embryonic tissues [1] and embryonic stem (ES) cells [2], absent in most normal adult cells and re-expressed in cancer (stem) cells [3–7]. HMGA2 utilizes its three AT-hook domains to bind to AT-rich sequences in the minor groove of DNA and this causes DNA conformational changes to facilitate transcriptional regulation [8, 9]. HMGA2 expression directly correlates with the level of malignancy and metastasis in different cancers [3, 10–12]. The expression of this oncofetal stem cell factor is regulated by the Lin28 - Let-7 pathway [13, 14]. Mutations to the 3′ untranslated region of the HMGA2 gene can impair the binding of microRNAs, including Let-7, miR142-3p, miR98 and miR-145 [15] and contribute to oncogenic transformation [16]. In breast tumors, increased Wnt/β-catenin signaling was shown to up-regulate HMGA2, promote EMT transformation, and increase tissue invasion of tumor cells [17].

Multi-functional HMGA2 has protective roles in ES and cancer cells. HMGA2 exhibits AP/dRP lyase activity and promotes base-excision repair (BER) under chemotherapeutic stress [18]. It also serves as chaperone to protect stalled replication forks from endonucleolytic collapse, thus, preventing DNA breaks and promoting replication restart [19]. HMGA2 interacts with both Ataxia telangiectasia mutated (ATM) and Ataxia telangiectasia and Rad3 related (ATR) kinases and is phosphorylated by ATM to increase cell survival under genotoxic stress [20, 21]. Upon DNA damage, HMGA2 increases cancer cell survival through prolonged phosphorylation of ATR and its downstream target checkpoint kinase 1 (CHK1) [21].

Mammalian telomeres are comprised of double stranded (ds) repetitive purine (A/G) rich ^5′^(TTAGGG) n^3′^ DNA repeats of 2-15 kb length and terminate in a 3′ single-stranded (ss) guanine (G)-rich overhang of 50-500 nucleotides [22]. Telomeres are protected by a complex of six telomere-associated shelterin members: TRF1/2, RAP1, TIN2, TPP1, and POT1. Telomeric repeat binding factor 2 (TRF2) is a key factor in telomere protection and chromosomal stability [23, 24]. The N-terminal TRF2 homo-dimerization domain (TRFH) and the C-terminal Myb-type DNA binding domain facilitate the binding of TRF2 homodimer to telomeric DNA [25, 26]. TRF2 is essential in forming and stabilizing the telomere (T) loop [27] which protects chromosomal ends from end-to-end fusions and blocks the activation of DNA repair mechanisms [23, 28, 29]. TRF2 serves as an interaction partner for shelterin members TRF1, RAP1, and TIN2; the latter connects the TPP1/POT1 complex to TRF1/2 and stabilizes DNA binding of TRF1 and TRF2 [30, 31].

TRF2 is a key inhibitor of DNA damage signaling at telomeres and does so by a two-step process of chromosomal protection [32]. In the absence of DNA damage, the TRFH domain of TRF2 binds to *Ataxia telangiectasia mutated* (ATM) surrounding residue S^1981^ of the ATM auto-phosphorylation site to inhibit the initial step of ATM-mediated DNA repair signaling at telomeres [33]. Independent of this ATM blocking function, the inhibitor of DNA damage repair (iDDR) region located within the C-terminal Hinge region of TRF2 can suppress DDR downstream of ATM, prevent telomeric deposition of 53BP1 and block telomere fusions [32]. TRF2 also interacts with the ATM downstream target checkpoint kinase 2 (CHK2) and locally represses CHK2 activation at telomeres by competing with ATM for binding to the S/TQ domain of CHK2 [34]. In response to genomic DNA damage, activated CHK2 phosphorylates residue threonine 188 (T188) located within the TRFH dimerization domain of TRF2, which triggers dissociation of TRF2^T188^ from telomeres to facilitate non-telomeric DNA damage repair [35, 36].

Here we report a novel protective function of HMGA2 at telomeres. We show that HMGA2 is localized at telomeres and interacts with TRF2, independently of the TRF2 interacting partner RAP1. The TRF2-HMGA2 protein interaction is independent of HMGA2-DNA binding, and unaffected by DNA damage. The telomere targeting drug KML-001 caused telomere-dysfunction induced foci (TIF) which were increased further with the knockdown (kd) of HMGA2. This dual telomere- and HMGA2-targeted treatment caused severe telomere dysfunction and genomic instability in cancer cells. This demonstrates the feasibility of the new therapeutic strategy in generating catastrophic genomic instability in HMGA2^+^ cancer cells by overcoming the telomere stabilizing function of HMGA2.

## RESULTS

### HMGA2 interacts with TRF2

**Figure 1:**
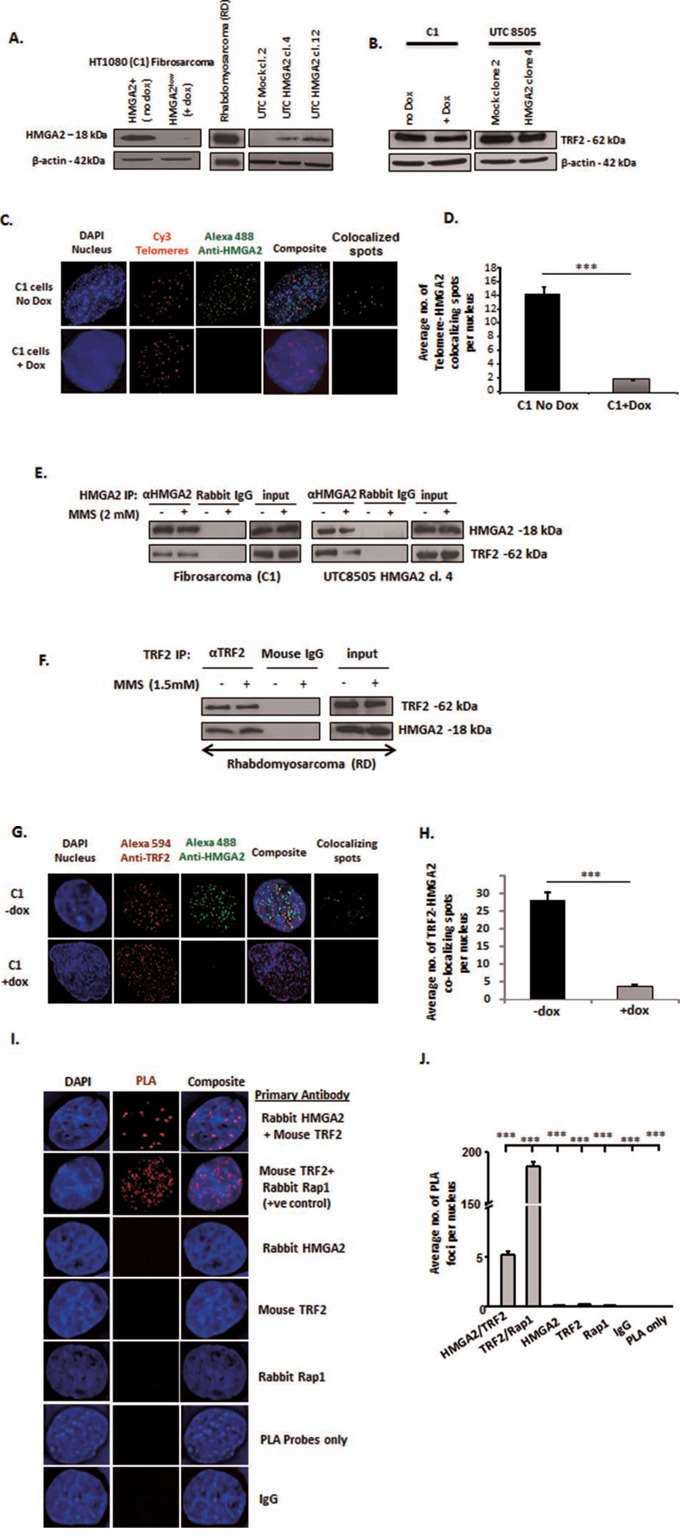
HMGA2 interacts with TRF2 **A.** Western blot showing HMGA2 expression in the three different cell models used in the study: endogenously HMGA2 expressing HT1080 (C1) Fibrosarcoma and Rhabdomyosarcoma (RD) and exogenous HMGA2-expressing transfectants of Undifferentiated Thyroid Cancer UTC8505. C1 fibrosarcoma cells contain a doxycycline (dox)-inducible shHMGA2 construct. Addition of dox resulted in the downregulation of HMGA2. **B.** Protein levels of TRF2 shown by Western blot in the endogenous and overexpressing producers of HMGA2 β-actin was used as loading control. **C.** Immuno-FISH showing HMGA2 localization at telomeres Blue-Nucleus; Red-Telomeres; Green-HMGA2; yellow-Telomere-HMGA2 colocalizing spots. **D.** A total of 50 nuclei were quantified and the graph shows the average number of Telomere-HMGA2 colocalizing spots per nucleus **E.** Interaction of HMGA2 with TRF2 +/−MMS is shown by HMGA2 co-immunoprecipitation (co-IP) from nuclear extracts of C1 and UTC8505 cells and **F.** by TRF2 co-IP in Rhabdomyosarcoma cells Appropriate IgG controls were employed. **G.** Double immunofluorescence displayed co-localization of TRF2 and HMGA2. Blue-Nucleus; Red-TRF2; Green-HMGA2; yellow-TRF2-HMGA2 co-localizing spots. **H.** A total of 50 nuclei were quantified and the graph shows the average number of TRF2-HMGA2 co-localizing spots per nucleus Doxycycline treated HMGA2 knock-down cells were used as negative control for HMGA2. **I.** Proximity Ligation Assay (PLA) confirmed the co-localization of TRF2 and HMGA2. Blue-Nucleus; Red-PLA foci. The well-known TRF2-RAP1 interaction was used as methodology positive control. Appropriate single primary antibodies, isotype control and PLA probes only were used as negative controls. **J.** A total of 30 nuclei were quantified and the graph shows the average number of PLA foci per nucleus Quantitative data are shown as mean +/− SEM; ***p<0.001.

In endogenous producers (HT1080/C1 fibrosarcoma transfectants with doxycycline (dox) regulated shHMGA2 expression and RD rhabdomyosarcoma cells) and the HMGA2 transfectants of undifferentiated thyroid carcinoma cells UTC8505, HMGA2 was exclusively detected in nuclear protein extracts. C1 cells showed a down-regulation of endogenous HMGA2 within 48h of dox treatment in Western blot (Fig. 1A) [18, 19, 21]. Changes in cellular HMGA2 levels had no effect on the TRF2 baseline protein expression levels (Fig. 1B, Suppl. Fig. 1). Combined immunofluorescence for HMGA2 and telomere FISH revealed localization of HMGA2 at telomeres in interphase nuclei (Fig. 1C). Dox treatment almost abolished these HMGA2 foci in C1 cells, indicating the specificity of this HMGA2 detection (Fig. 1C). We observed on average fourteen HMGA2-telomere co-localizing foci per nucleus in HMGA2^+^cells vs. 1-2 foci in HMGA2^low^ dox treated cells, confirming that HMGA2 knockdown was almost complete at telomeres (Fig. 1D). Co-IP of HMGA2 resulted in the specific pulldown of TRF2 in nuclear protein extracts of C1 and UTC8505 transfectants (Fig. 1E) and reverse co-IP with TRF2 resulted in the detection of HMGA2 (Fig. 1F), demonstrating the interaction of HMGA2 with the key shelterin protein TRF2. Treatment with the DNA alkylating agent methyl methanesulfonate (MMS) had no effect on this interaction (Fig. 1E, F). We assessed the specificity of the antibodies used in our co-IP studies using specific RNAi mediated knockdown (kd), followed by pulldown experiments. Upon RNAi mediated TRF2 kd, IP and subsequent Western blot detection with the antibody to human TRF2 failed to detect TRF2 (Suppl. Fig. 1A). In addition, we were unable to detect the TRF2 interaction partner RAP1 used as positive control in the TRF2 co-IP studies (Suppl. Fig. 2A). When the TRF2 antibody was used for co-IP on dox-treated HMGA2^low^ C1 cells, the HMGA2 antibody failed to detect HMGA2 in the IP despite the fact that RAP1 was detectable. These results validated the specificity of the HMGA2 antibody used (Fig. 1A, Suppl. Fig. 2B) and indicate successful co-IP of TRF2 and RAP1. The important fact that we were unable to co-IP the single strand telomeric DNA binding shelterin member POT1 with HMGA2 indicated the specificity of the newly discovered interaction of HMGA2 with TRF2 (data not shown). Of note, dox treatment specifically downregulated HMGA2 but failed to interfere with the expression of HMGA1, indicating that the biological effects observed were specific to HMGA2 (Suppl. Fig. 3A, B, C).

Using co-immunofluorescence imaging, we identified co-localizing foci of HMGA2 and TRF2 in interphase nuclei of cancer cells (Fig. 1G). On average, 27 co-localized foci per nucleus were observed in HMGA2^+^ C1 versus a negligible number of co-localized spots upon HMGA2 kd (Fig. 1H), confirming the TRF2-HMGA2 interaction we detected by co-IP. To probe the co-localization of HMGA2 with TRF2 further, we employed a proximity ligation assay (PLA), which is a highly sensitive protein co-localization technique capable of detecting two proteins ≤ 40 nm apart [37] (Fig. 1I). PLA confirmed the known interaction between TRF2 and RAP1 used as positive control. We detected on average five HMGA2-TRF2 PLA foci per nucleus, whereas 0-1 PLA foci were detected with the individual antibodies, non-immune IgG isotypes and the PLA probe only were used as negative controls (Fig. 1J).

### AT-hooks and linker (L1) region contribute to the HMGA2-TRF2 interaction

**Figure 2:**
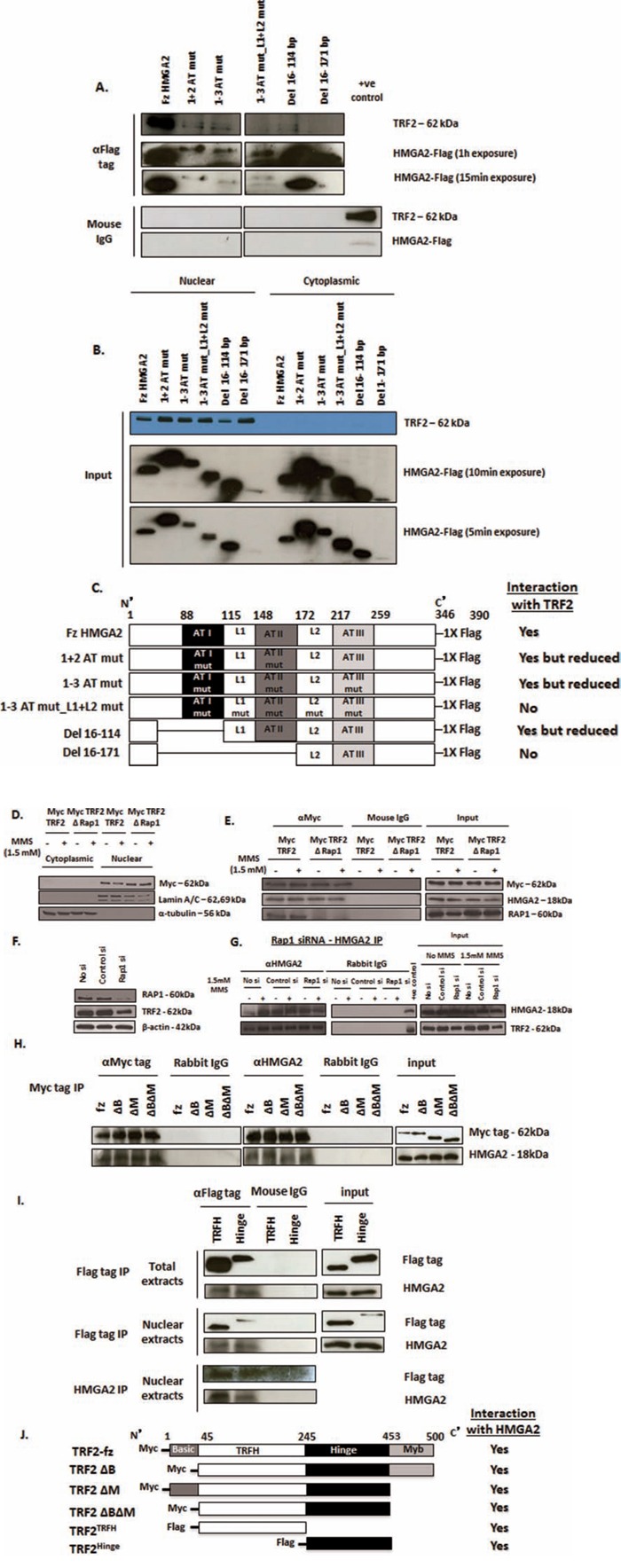
Structural determinants of HMGA2-TRF2 interaction **A.** HMGA2 structural determinants contributing to the interaction with TRF2 as determined by immunoprecipitation studies upon transient expression in HEK293T of Flag-tagged AT-hook mutants (*1+2 AT mut; 1-3 AT mut*), linker mutant (*1-3 AT mut_L1+L2 mut*) and N-terminally truncated constructs (*Del 16-114 and Del 16-171*) of HMGA2. **B.** Western Blot showing exclusive nuclear localization of TRF2 whereas the HMGA2 mutants were expressed in both the nuclear and cytoplasmic compartments. A, **C.** Strong interaction with TRF2 was observed for fzHMGA2 and at a significantly lower level with both HMGA2 AT-hook mutants and the Del 16-114 mutant lacking AT-hook 1. Interaction with TRF2 was not observed when the linker L1 was mutated along with AT-hook 2 (*1-3 AT mut_L1+L2 mut*) or deleted (*Del 16-171*). **C.** Schematic representation of the Flag-tagged AT-hook, linker mutants and the truncated constructs of HMGA2 with the corresponding co-IP results for the interaction with TRF2. **D.** Western Blot showing nuclear localization of both the Myc tagged full size TRF2 and RAP1 binding domain deleted TRF2 (ΔRap1) constructs. Lamin a/c was used as nuclear marker and α-tubulin was used as cytoplasmic marker. **E.** Nuclear extracts were prepared from RD cells transiently transfected with Myc tagged full size TRF2 and RAP1 binding domain deleted TRF2 mutant. Myc tag IP was performed. Both the full size TRF2 and RAP1 binding domain deleted TRF2 showed interaction with HMGA2 in the presence and absence of MMS. **F.** Western blot of RAP1 siRNA-mediated specific knockdown of RAP1 also showed slightly reduced protein expression of TRF2. β-actin was used as loading control. **G.** Nuclear extracts prepared from RAP1 knocked down cells were treated with and without MMS. HMGA2 IP was then performed that showed interaction of HMGA2 with TRF2 despite RAP1 knockdown. Appropriate IgG controls were employed. **H, I.** Myc tagged TRF2 constructs (full size; fz, ΔB, ΔM, ΔBΔM) and FLAG tagged TRF2 constructs (TRFH and Hinge) were transiently transfected into C1 fibrosarcoma cells. After 48h, nuclear and total extracts were prepared and processed for co-IP. **H.** co-IP using Myc tag on TRF2 demonstrated interaction of all four Myc tagged TRF2 constructs with HMGA2. **I.** co-IP with FLAG tag on TRF2 from total and nuclear extracts and reverse co-IP with HMGA2 were performed all of which demonstrated interaction of HMGA2 with the TRFH and hinge domains of TRF2. Appropriate IgG controls were employed. **J.** Schematic representation of the truncated constructs of TRF2 with Myc and Flag tags and their HMGA2 interaction as shown by co-IP.

The three multi-functional AT-hook motifs facilitate the binding of HMGA2 to AT-rich regions within the minor groove of DNA, contain a nuclear localization signal, and possess lyase activity [8, 18]. We generated FLAG-tagged HMGA2 mutants in which (i) all lysine and arginine residues within the first and second or in all three AT-hooks were mutated to alanine and (ii) alanine mutations were made to the linker regions L1 and L2 (Suppl. Fig. 4A). These AT1+2/AT1-3 HMGA2 mutants had been reported not to bind to genomic DNA [8, 18]. Upon transient transfection into HEK293T cells, both AT-hook mutants tested (1+2 AT mut and 1-3 AT mut) were still able to co-IP TRF2, albeit at significantly lower amount (Fig. 2A). We then generated a FLAG-tagged 1-3 AT HMGA2 mutant with additional alanine mutations to both linkers, L1 and L2 (1-3 AT mut_L1+L2 mut) (Fig. 2C). Transient transfection into HEK293T followed by anti-FLAG co-IP did not show an interaction with TRF2 (Fig. 2A). To further investigate whether the AT-hooks and/or linker regions (L1 and/or L2) were responsible for this protein interaction with TRF2, we generated Flag-tagged expression constructs with N-terminal deletion of just the AT-hook I (Del 16-114) or deletion of AT-hooks I and II and the linker L1 (Del 16-171). Anti-FLAG co-IP revealed a positive interaction of TRF2 with Del 16-114 mutant but at markedly reduced levels, whereas the Del 16-171 mutant consistently failed to interact with TRF2 (Fig. 2A). This latter result indicated that L2, AT-III or the C-terminal region of HMGA2 did not contribute to the interaction with TRF2. L1 alone and the region of HMGA2 encompassing L1 and AT-II facilitated a weak HMGA2-TRF2 interaction (Fig. 2C). Taken together, the IP results obtained from all mutant constructs tested suggested that most efficient interaction between HMGA2 and TRF2 required AT-hooks I+II plus the L1 linker region (Fig. 2C). The inputs of Fig. 2A are depicted in Fig. 2B and nuclear extracts were used in Fig. 2A. DNAse digest of genomic DNA did not diminish the HMGA2-TRF2 interaction (Suppl. Fig. 4B, C). To test whether FLAG-tagged 1+2ATmut and 1-3ATmut proteins were still able to localize to telomeres, we performed combined immunofluorescence for FLAG and telomere FISH in dox-treated C1 cells upon transient transfection with fzHMGA2, 1+2 ATmut and 1-3ATmut expression constructs. The AT-hook mutants were still able to co-localize to telomeres (Suppl. Fig. 5A, B). This suggested that these HMGA2 mutant proteins may utilize additional unknown means to ensure telomeric localization and did not solely rely on the interaction with TRF2 (Fig. 2A, 2C), which would imply a more complex interaction of HMGA2 at telomeres.

### HMGA2 interacts with two specific TRF2 domains

Human TRF2 is composed of an N-terminal basic domain, a TRF homo-dimerization domain (TRFH), a hinge region containing binding sites for RAP1 and TIN2, and a C-terminal Myb-like DNA-binding domain [25, 26]. RAP1 is recruited to telomeres by binding to TRF2 [38]. To determine if RAP1 was required for the HMGA2-TRF2 interaction, we utilized a TRF2 mutant construct with the RAP1 binding site deleted (TRF2ΔRAP1) (Fig. 2D) [38]. We confirmed that the expression of Myc tagged TRF2ΔRAP1 fusion protein was exclusively in the nucleus and not affected by MMS treatment (Fig. 2D). Although TRF2ΔRAP1 mutant was unable to bind RAP1, co-IP revealed the interaction with HMGA2 and MMS treatment had no effect on the interaction of HMGA2 with native TRF2 or TRF2ΔRAP1 (Fig. 2E). We verified these results using a specific RNAi mediated kd of RAP1. RAP1 kd reduced but did not knockdown TRF2 expression (Fig. 2F). Following RAP1 kd, HMGA2 co-IP revealed the presence of TRF2, indicating again that the interaction between HMGA2 and TRF2 is independent of RAP1 (Fig. 2G). Next, we expressed human Myc-tagged TRF2 mutant constructs with deletion of the basic domain (TRF2ΔB), the Myb-like DNA binding domain (TRF2ΔM), and dual deletion (TRF2ΔBΔM) (Fig. 2H)[39]. Co-IP with the anti-Myc antibody showed that all three Myc-tagged TRF2 mutant proteins were able to interact with HMGA2 (Fig. 2H). Since the TRFH and hinge regions were common to all TRF2 mutant constructs tested (Fig. 2J), we tested by co-IP the ability of FLAG-tagged constructs of both TRF2 molecular regions to interact with HMGA2 [40]. When expressed in C1 cells, both TRF2 mutant proteins were at least partially expressed in the nucleus (Fig. 2I). Anti-FLAG co-IP studies revealed that the individual TRFH and hinge region of human TRF2 were each able to interact with HMGA2 (Fig. 2I, 2J).

### HMGA2 has a novel role in telomere end protection

**Figure 3:**
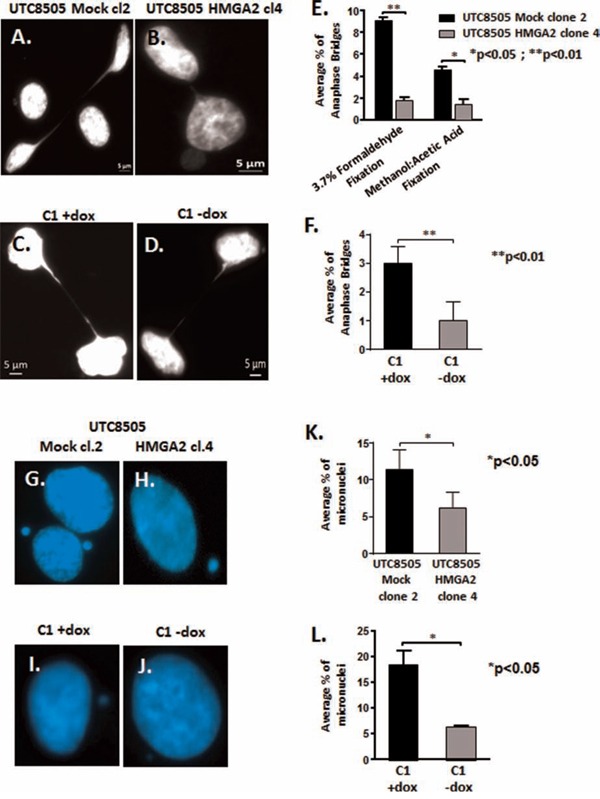
HMGA2 reduces telomere instability Anaphase bridges and micronuclei were quantified in three independent experiments (300 nuclei analyzed per experiment) and the average percentage of bridges and micronuclei per total of 900 analyzed nuclei is shown. Representative images of anaphase bridges in **A.** UTC8505 Mock clone 2, **B.** UTC8505 HMGA2 clone 4, **C.** C1+Dox and **D.** C1-No Dox fixed with 3.7% formaldehyde. **E.** Quantification of the average percentage of anaphase bridges in UTC8505 Mock clone 2 and HMGA2 clone 4 under two different fixatives and **F.** in C1 fibrosarcoma −/+ Dox fixed with 3.7% formaldehyde Representative images of micronuclei in **G.** UTC8505 Mock clone 2, **H.** UTC8505 HMGA2 clone 4, **I.** C1+Dox and **J.** C1-No Dox Quantification of the average percentage of micronuclei **K.** in UTC8505 and **L.** in C1 −/+ Dox Quantitative data are shown as mean +/− SEM; *p<0.05, **p<0.01.

We had shown previously that loss of HMGA2 promotes increased genomic instability and this coincided with the occurrence of dicentric chromosomes in cancer cells, which are the result of impaired telomere end protection [19, 41]. Telomeric dysfunction can lead to impaired chromosomal segregation by initiating repeated Breakage-Fusion-Bridge (BFB) cycles that generate anaphase bridges and micronuclei [42–44]. To assess the role of HMGA2 in chromosomal instability, we compared HMGA2^negative^ UTC8505 mock cells (Fig. 3A) with HMGA2 over-expressing UTC8505 transfectants (Fig. 3B) or dox-treated HMGA2^low^ C1 cells (Fig. 3C) with endogenous HMGA2^+^ C1 cells (Fig. 3D). Intriguingly, the presence of HMGA2 resulted in a significantly reduced percentage of anaphase bridges in both cancer cell models (Fig. 3E, 3F). To determine the presence of anaphase bridges, cells were fixed with either 3.7% formaldehyde (Fig. 3E) or methanol:acetic acid (Suppl. Fig. 6A, B). Both fixation methods confirmed that the formation of anaphase bridges was reduced significantly in the presence of HMGA2. Similar results were obtained for micronuclei in UTC8505 mock (Fig. 3G) vs. UTC8505-HMGA2 transfectants (Fig. 3H) and HMGA2^low^ C1 (Fig. 3I) vs. HMGA2^+^ C1 cells (Fig. 3J). The percentage of micronuclei was found to be significantly decreased in the presence of HMGA2 in both cancer cell models (Fig. 3K, 3L). Next, we performed telomere FISH on anaphase bridges to determine if they contained telomeric DNA. We detected telomere signals on anaphase bridges confirming previous reports [45, 46] that the chromatin bridges formed upon Breakage-Fusion-Bridge cycles contained telomere sequences (Suppl. Fig. 6C). Similar to previous reports, [45, 46], we detected telomere signals in approx. 60% of bridges with no significant differences in both −/+ dox treated C1 cells, suggesting that cellular HMGA2 did not affect the telomere composition of the anaphase bridges (data not shown).

### HMGA2 affects telomere architecture and defines telomere signatures in cancer cells

**Figure 4:**
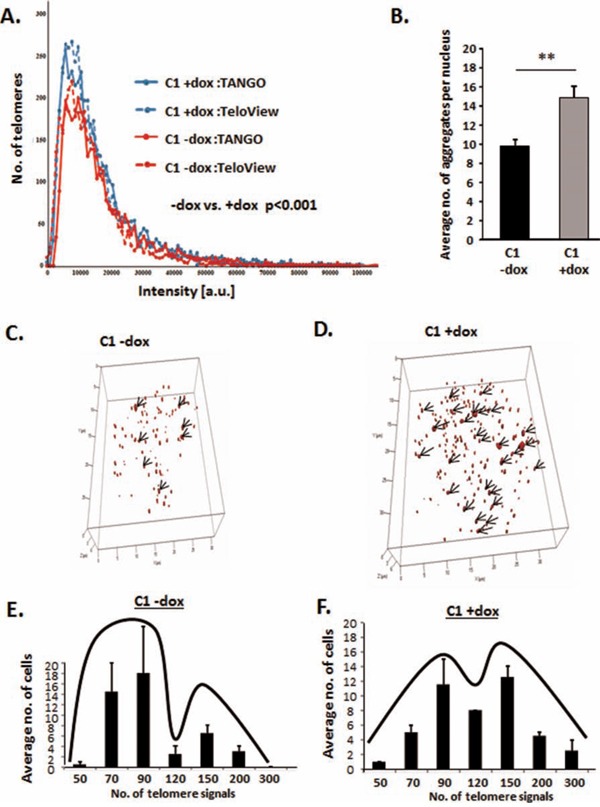
HMGA2 affects telomere architecture and defines telomere signatures in cancer cells Telomere FISH of interphase nuclei showing Dox untreated HMGA2^high^ cells with **A, E.** fewer telomere signals and **B, C.** fewer telomere aggregates and dox treated HMGA2^low^ cells with **A, F.** increased telomere signals and **B, D.** increased telomere aggregates as analyzed by TeloView software. **A.** Quantitative analysis with both TeloView and TANGO software is shown for an average of 50 individual nuclei. Two independent experiments were performed. Telomere signal intensities in 50 nuclei were counted and showed statistical significance (***p<0.001) in −/+ dox treated C1 cells. **C, D.** 3D representation of the telomeric aggregates in a single nucleus −/+ Dox **E, F.** Single cell 3D telomere analysis was performed on 50 nuclei for each group and nuclei were categorized according to the number of telomere signals. Quantitative data are shown as mean +/− SEM; **p<0.01.

Having shown that HMGA2 diminishes telomere-mediated genomic instability in human cancer cells, we wanted to determine if the loss of this telomere protective role of HMGA2 involves changes in 3D telomere architecture [47–49]. For these studies, we used C1 cells +/− dox to regulate the level of endogenously produced HMGA2. Although HMGA2^low^ C1 cells contained an increased number of telomere signals, the distribution of average telomere fluorescence intensities was independent of HMGA2 (Fig. 4A). Telomere data were analyzed using two different analysis software packages, which gave similar results (Fig. 4A)[47, 50, 51]. In HMGA2^low^ C1 cells vs. HMGA2^+^ cells, the enhanced number of telomere signals coincided with a significant increase in the number of telomere aggregates indicating that loss of HMGA2 coincided with increased telomere instability [47](Fig 4B-4D). Employing single cell analysis, we determined the effect of HMGA2 on the number of telomere signals per nucleus in individual cancer cells. We observed a shift towards a cell population with a higher number of telomere signals in C1 cells with diminished cellular HMGA2 (Fig. 4E, 4F). We concluded that HMGA2 reduced telomere instability in cancer cells.

### HMGA2 protects against telomeric TRF2 depletion and TIF formation

**Figure 5:**
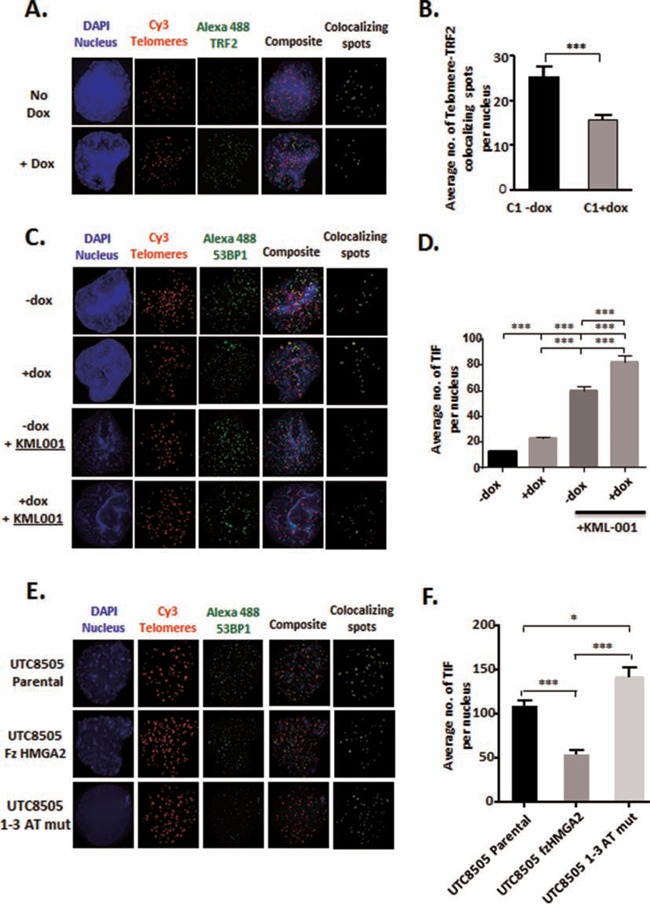
HMGA2 protects against telomeric TRF2 depletion and TIF formation **A.** Telomeric localization of TRF2 was determined in C1 cells by Immuno-FISH by co-staining for TRF2 with telomeres in the presence (−dox) and absence (+dox) of HMGA2. Blue-Nucleus; Red-Telomeres; Green-TRF2; yellow- Telomere-TRF2 co-localizing spots. **C.** Telomere Dysfunction-Induced Foci (TIF) were evaluated by Immuno-FISH where 53BP1 was co-stained with telomeres in C1 cells in the presence (−dox) and absence (+dox) of HMGA2 and following induction of telomere damage with KML001. Blue-Nucleus; Red-Telomeres; Green-53BP1; yellow-Telomere-53BP1 co-localizing foci. **E.** TIF were also determined following stable transfection of fz and AT-hook mutant HMGA2 in UTC8505 parental cells. **B.** Average number of TRF2 signals per nucleus co-localizing with telomeres were quantified and graphed. Quantification of the average number of telomere-53BP1 co-localizing foci (TIF) per nucleus are shown **D.** under HMGA2 knock-down, upon challenge with the telomere damaging agent KML001 and **F.** upon stable transfection with fz and AT-hook mutant HMGA2. A total of 50 nuclei were counted and quantitative data are shown as mean +/− SEM; * p<0.05; ***p<0.001.

TRF2-depleted telomeres initiate the recruitment of DNA damage response (DDR) factors, including 53BP1, which participates in the formation of telomere dysfunction induced foci (TIF) [24, 52], activates cell cycle checkpoints and promotes telomere end-to-end fusions [23, 53]. We hypothesized that HMGA2 contributes to the protection of telomeres by securing TRF2 at telomeres and, thus prevents the formation of TIF in the absence of telomere damage. Indeed, diminished cellular HMGA2 levels coincided with an approx. 40% reduction in TRF2 foci localized at telomeres (Fig. 5A, 5B). Next, we determined the number of TIF as reflected by 53BP1 foci localized at telomeres. HMGA2 kd resulted in an approx. 50% increase in the number of TIF (Fig. 5C, 5D). While these HMGA2-induced telomeric changes occurred in the absence of telomeric damage, we determined TIF in the presence of the telomere-targeting arsenite drug KML-001 to assess whether HMGA2 can aid in the protection of telomeres under telomere-specific stress [54]. HMGA2 kd significantly increased the sensitivity of C1 cells towards KML-001 indicating a protective role of HMGA2 against the telomere-damaging effects of KML-001 between 3 and 10 μM (Suppl. Fig. 7). This corresponded to markedly increased TIF numbers upon KML-001 treatment, which was enhanced further with cellular HMGA2 levels diminished (Fig. 5C, 5D). We concluded that HMGA2 can protect against the actions of telomere-targeting chemotherapeutic drugs in cancer cells. To assess the role of the AT-hooks in telomere protection, we determined TIF in HMGA2-negative UTC8505, which had been stably transfected with HMGA2 constructs encoding full size (fz) and 1-3 ATmut. TIF formation was reduced by 50% in the fzHMGA2 transfectants when compared to the HMGA2-negative parental UTC8505 cells (Fig. 5E, 5F). By contrast, 1-3 ATmut HMGA2 showed significantly higher TIF numbers than parental UTC8505 and three fold more TIF than fzHMGA2 clones (Fig. 5E, 5F), implicating the AT-hooks of HMGA2 in telomere stability. This telomere-protective role of HMGA2 was observed in a diverse set of tumor cells, including C1 fibrosarcoma, UTC8505 thyroid cancer, and U251 glioblastoma cells upon siHMGA2 mediated silencing (Suppl. Fig. 8).

### HMGA2 silencing reduces telomeric DNA bound to TRF2

**Figure 6:**
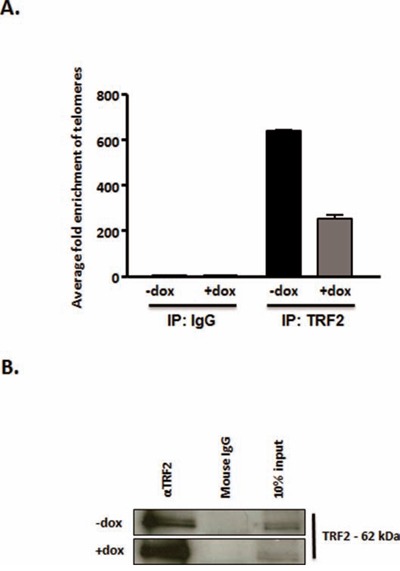
HMGA2 silencing reduces telomeric DNA bound to TRF2 Telomere-Chromatin immunoprecipitation was performed with TRF2 antibody in the presence of HMGA2 (−dox) and at HMGA2low conditions (+dox). The presence of telomeric DNA was quantified by real time quantitative PCR in TRF2 immunoprecipitated and purified DNA [73]. Enrichment of telomeric DNA in telomere-ChIP upon TRF2 IP was normalized to the corresponding IgG control. **A.** A significant reduction in fold enrichment of telomeric DNA was observed upon knockdown of HMGA2 (+dox) p<0.001***. **B.** TRF2 detection, in the corresponding ChIP samples, by Western blot following TRF2 pull-down is shown compared to IgG control. Quantitative data are shown as mean +/− SEM; ***p<0.001.

We hypothesized that a destabilization of TRF2 at telomeres at decreased cellular HMGA2 levels would result in reduced amount of TRF2 bound to telomeric DNA. To test this, we performed telomere-ChIP assays. We quantified by telomere-specific real time quantitative PCR the amount of telomere DNA (Fig. 6A) bound to TRF2 that had been immunoprecipitated in C1 cells with normal levels of HMGA2 (−dox) and upon HMGA2 kd (+dox) (Fig. 6B). Corresponding IgG served as IP control and failed to pull down TRF2 (Fig. 6B). Decreased HMGA2 (+dox) resulted in a 3-fold reduced amount of telomere DNA bound to TRF2 (Fig. 6A). These ChIP results are consistent with our immunoFISH data, which showed significantly reduced TRF2 signals at telomeres under HMGA2 depleted conditions (Fig. 5A, 5B).

### HMGA2 affects the phosphorylation of TRF2 and the telomeric signaling cascade

**Figure 7:**
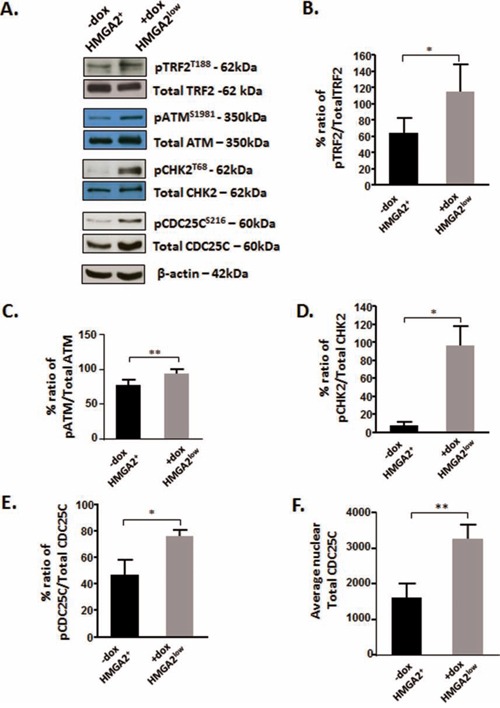
HMGA2 affects the phosphorylation of TRF2 and the telomeric signaling cascade **A.** Representative Western blots are shown for the detection of phosphorylated TRF2, ATM, CHK2 and CDC25C in C1 cells under high and low HMGA2 status (−/+dox). Densitometric analyses of the phospho proteins relative to the total proteins were done and graphed for **B.** TRF2; **C.** ATM; **D.** CHK2 and **E.** CDC25C. **F.** The average total CDC25C was quantified from nuclear protein fractions. β-actin was used to control for equal loading of proteins. Quantitative data are shown as mean +/− SEM; *p<0.05, **p<0.01.

Phosphorylated TRF2^T188^ is displaced from telomeres and recruited to genomic DNA damaged sites [34–36]. Telomeric loss of TRF2 initiates a telomere specific signaling pathway which includes ATM, its downstream target CHK2 (checkpoint kinase 2) and CDC25C (cell division cycle 25C) [55]. We showed in HMGA2^low^ cells that the loss of TRF2 at telomeres coincided with a significant increase in pTRF2^T188^ levels (Fig. 7A, 7B). HMGA2 kd also resulted in markedly enhanced levels of activated pATM^S1981^ and pCHK2^T68^ (Fig. 7A, 7C, 7D), indicating that nuclear HMGA2 can modulate the phosphorylation and activity status of the TRF2-ATM-CHK2 signaling axis. Next, we studied the dual phosphatase CDC25C, which is a key regulator of mitotic entry and specific telomere damage target [55, 56]. Loss of TRF2 at telomeres was shown to cause the phosphorylation of CDC25C at residue S216, which triggers the cytoplasmic export and degradation of pCDC25C^S216^ [55, 57, 58]. HMGA2 kd resulted in a significant increase in pCDC25C^S216^ and nuclear total CDC25C (Fig. 7A, 7E, 7F). We concluded that HMGA2 affects TRF2 occupancy at telomeres and modulates the activity status of the ATM-CHK2-CDC25C signaling axis.

In summary, we identified novel roles of HMGA2 in maintaining TRF2 occupancy at telomeres and preventing telomere dysfunction-induced genomic instability in HMGA2^+^ cancer cells.

## DISCUSSION

Although an association between HMGA2 and telomeres during metaphase was observed previously [59], the role of this oncofetal protein at telomeres has remained elusive. In the present study, we have identified HMGA2 as a new interaction partner of the key shelterin member TRF2 at telomeres of interphase nuclei and demonstrated a novel role of HMGA2 in telomere protection in human cancer cells. Silencing of endogenous HMGA2 alone was sufficient to increase telomere dysfunction–induced foci (TIF) and the formation of micronuclei and anaphase bridges in cancer cells, indicating a protective function of HMGA2 in telomere stability. Anaphase bridges occur as a result of dysfunctional telomeres undergoing end-to-end chromosomal fusions [41] and initiate Breakage-Fusion-Bridge (BFB) cycles [42] which contribute to the hallmarks of cancer, aneuploidy and genome instability [43]. We had previously reported cytogenetic alterations in chromosomal metaphase spreads indicating telomere instability upon knockdown of HMGA2, independent of any genotoxic drugs [19]. The telomere-protective role of HMGA2 was confirmed in a human thyroid cancer cell model devoid of endogenous HMGA2 where the introduction of exogenous HMGA2 increased telomere stability. Remarkably, HMGA2 also reduced telomere dysfunction caused by the telomere-targeting drug KML-001 [54, 60]. By contrast, the general genomic DNA alkylating agent methyl methanesulfonate (MMS) failed to significantly affect TIF numbers (data not shown), which may be due to the inability of MMS to destabilize telomeres. To further investigate the telomeric role of HMGA2 in cancer cells, we performed 3D interphase telomere analysis to quantify telomere number and aggregate formation. Telomere aggregates occur with telomere instability and are independent of telomerase activity [47]. HMGA2 silencing had no effect on telomere length distribution but increased the number of telomere aggregates, reflecting increased telomere stress in HMGA2^low^ cells. Importantly, HMGA2 depletion promoted the occurrence of novel cancer cell subpopulations with higher telomere numbers, suggesting the evolution of new HMGA2^low^ genomic phenotypes fueled by telomere dysfunction with end-to-end telomere fusions followed by repeated BFB cycles [47].

We identified TRF2, the key shelterin member responsible for telomere integrity, as a new interaction partner of HMGA2. On average 14.5% of the detected telomere signals co-localized with HMGA2 foci and PLA assays confirmed the HMGA2-TRF2 interaction with a lower number of foci. Although the inherent architectural properties of HMGA2 may influence the HMGA2-TRF2 interaction [61], we cannot exclude antibody-epitope kinetics to negatively affect PLA detection. Functionally relevant interactions with TRF2 at low incidence have been reported for other TRF2 interaction partners, including Mre11/Rad50/Nbs1 [62], XPF/ERCC1 [63] and Cockayne syndrome group B protein [40].

The HMGA2-TRF2 interaction occurred independent of genomic DNA. Our mutation analysis revealed that the N-terminal ATI-L1-ATII region of HMGA2, but not the L2-ATIII-C-terminal region, was required for TRF2 pulldown. While HMGA2 constructs with intact L1 linker alone were able to weakly interact with TRF2, only HMGA2 with intact ATI-L1-ATII region was most effective in pulldown of TRF2, suggesting a more complex multi-domain mediated interaction. The TRF2 Myb DNA binding domains and the RAP1 binding motif located within the hinge region (aa 286-299) were dispensable for the interaction with HMGA2. Thus, the TRF2-HMGA2 interaction did not depend on TRF2 DNA-binding or TRF2-RAP1 heterodimerization. Of importance is the fact that despite its interaction with TRF2 and unlike wild type HMGA2, AT hook mutant HMGA2 was unable to rescue TIFs. This suggested an essential role for AT hooks in telomere protection either through telomeric DNA-binding or other AT hook functions such as AP/dRP-lyase activity. We currently cannot exclude that the AT hook-dependent DNA repair function of HMGA2 plays a role for the stabilization of TRF2 at telomeres.

Both, the TRF homology (TRFH) domain and hinge region of TRF2 were capable of independently interacting with HMGA2. These two TRF2 domains are involved in a two-step protective mechanism to ensure TRF2-mediated chromosomal end protection [32]. The TRFH domain of TRF2 is a direct binding site of ATM, and TRF2 is critical for blocking the activation of ATM and its downstream target CHK2 to inhibit telomere mediated DNA damage response (DDR)[32-34, 64]. Intriguingly, HMGA2 specifically interacts with both TRF2 and ATM [20]. The interaction of HMGA2 with TRFH did not involve Y/FxLxP related motifs known to be a preferred target motif for the TRFH domain of TRF2 [65, 66] since this motif is completely absent in HMGA2.

The hinge region of TRF2 is composed of three interacting motifs for RAP1 (aa 286-299), TIN2 (aa 352-367) and a region participating in the inhibition of DDR (iDDR; aa 407-431). The latter independently prevents telomere recruitment of the ubiquitin ligase RNF168 which facilitates the formation of 53BP1-containing TIF, non-homologous end joining (NHEJ), telomere aggregation and chromosomal fusions [32]. While we cannot rule out a participation of the TIN2 motif in the interaction between TRF2 and HMGA2, our functional studies revealed significantly reduced numbers of 53BP1-containing TIF in the presence of HMGA2.

DNA damage and/or telomere shortening induce ATM mediated phosphorylation of both CHK2 at residue T68 and TRF2 in its TRFH domain at T188 (pTRF^T188^); the latter results in the dissociation of TRF2 from telomeres [35, 36]. Here we demonstrated for the first time an involvement of HMGA2 in this telomere signaling pathway. In the absence of telomere damaging agents, silencing of HMGA2 alone was sufficient to (i) reduce TRF2 foci at telomeres, (ii) cause functional readouts of telomere dysfunction and (iii) increase the levels of pATM^S1981^, pTRF2^T188^ and pCHK2^T68^. Importantly, HMGA2 knockdown did not reduce total cellular TRF2 protein levels in cancer cells indicating that this telomere dysfunction signaling phenotype was specific to the HMGA2 knockdown and associated with reduced telomeric localization of TRF2. This was supported further by telomere-ChIP assay, which revealed a significantly reduced amplification of telomere DNA upon IP of TRF2 in dox-treated (HMGA2^low^) C1 cells.

Our results identified HMGA2 as a novel negative modulator of the ATM dependent signaling pathway at telomeres and provided functional evidence for the additional telomere- and genome-protective role that cancer (stem) cells benefit from when they express HMGA2. Our results suggest that HMGA2, by binding to ATM and TRF2, promotes TRF2 occupancy at telomeres and increases the threshold for telomere damage signaling. Taken into consideration other known protective functions of HMGA2 in for example DNA damage repair and replication fork stabilization [4, 5, 18, 19], our findings revealed an as yet less known protective mechanism facilitating enhanced chemoresistance of HMGA2^+^ cancer cells to the telomere-damaging drug KML-001.

HMGA2 silencing and the corresponding increase in TIF coincided with slower proliferation of cancer cells. We focused at the dual phosphatase CDC25C, a key factor of mitotic entry. Telomere stress triggers the activation of the telomere specific ATM-CHK2-CDC25C signaling pathway, which can initiate G2/M arrest depending on p53 status [55, 67]. Nuclear CDC25C activity is extensively regulated by post-translational modifications that involves ATM-CHK2 mediated CDC25C phosphorylation at serine residue S216 and this leads to G2/M arrest [68]. Silencing of HMGA2 resulted in a significant increase in both nuclear pCDC25C^S216^ and, unexpectedly, total CDC25C. The hitherto unknown ability of HMGA2 to regulate nuclear CDC25C content independent of pCDC25C^S216^ status requires further investigations and may be clinically relevant as low cellular HMGA2 levels promote the emergence of cancer cell populations with heterogeneous telomere phenotypes.

**Figure 8:**
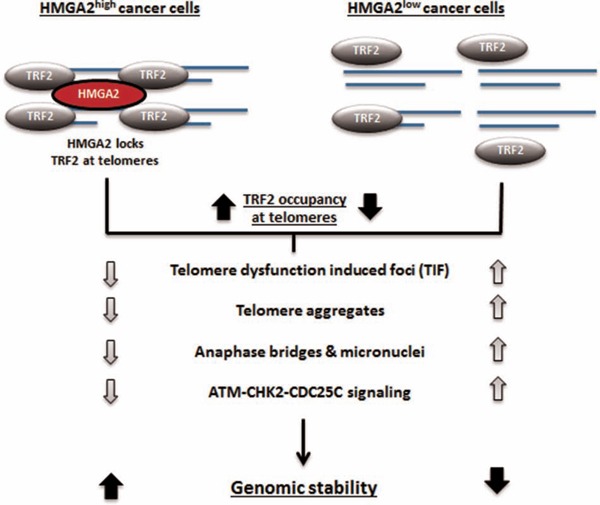
Schematic illustration of the proposed interaction of HMGA2 with TRF2 at telomeres and the resulting functional consequences for telomere stability

We have summarized the major findings of this paper in Fig. 8. HMGA2 stabilizes TRF2 at telomeres involving the AT hook function and the binding to TRF2. The ability of HMGA2 in cancer (stem) cells to affect the stability of interphase telomeres and increase the resistance to telomere-targeting drugs identifies HMGA2 as an attractive novel therapeutic target to induce lethal telomere-mediated genomic instability in HMGA2^+^ cancer cells.

## MATERIALS AND METHODS

### Cell culture

Endogenously HMGA2 expressing Rhabdomyosarcoma (RD) and HT1080 fibrosarcoma (C1), over-expressing HMGA2 transfectants of the undifferentiated thyroid cancer UTC8505 (Mock and HMGA2 clone 4) were employed in the study (Natarajan et al., 2013). C1 cells were generously provided by Dr. Peter Dröge, Nanyang Technological University, Singapore [18, 19]. HEK293T cells were employed for transient transfections. The cell lines were cultured in DME-F12 1:1 (Thermo Scientific, Ottawa, ON) supplemented with 10% fetal bovine serum (FBS; Sigma, Oakville, ON) and maintained at 5% CO_2_ in a 37°C humidified incubator. HMGA2 expressing UTC8505 transfectants were cultured with 500 μg/ml geneticin (Life Technologies, Burlington, ON). HT1080-C1 fibrosarcoma cells stably expressing a lentiviral shHMGA2 construct under the control of the doxycycline (dox) promoter were cultured under 3μg/ml puromycin (Sigma) selection. Endogenous HMGA2 expression in C1 transfectants was significantly down-regulated following exposure to 4μg/ml of dox (Sigma) for 4 days. DNA damage was induced using methyl methanesulfonate (MMS, Sigma) and exclusive telomeric damage was induced using sodium (meta-)arsenite (KML001, Sigma).

### Plasmid constructs

Myc tagged TRF2, Myc TRF2 ΔRAP1, Myc TRF2 ΔB, Myc TRF2 ΔM and Myc TRF2 ΔBΔM constructs were kind gifts from Dr. Titia De Lange, Rockefeller University, NY, USA. Flag tagged TRFH and Hinge constructs were kind gifts from Dr. Xu-Dong Zhu, McMaster University, Hamilton, ON. Synthetic gene constructs of AT-hook and Linker mutants of HMGA2 with lysine, arginine, glutamine and glutamic acid residues replaced with alanine [8, 18], and containing a C-terminal FLAG tag were manufactured by Life Technologies.

### Plasmid DNA transient transfection

Cells (1×10^6^) were seeded in a 100 mm petri dish and grown overnight at 5% CO_2_ in a 37°C humidified incubator. Plasmid DNA (2 μg) was transiently transfected into cells for 48h using Effectene transfection reagent (Qiagen, Toronto, ON) according to the manufacturer's instructions.

### Plasmid DNA stable transfection

UTC8505 parental cells (1×10^6^) were seeded in a 100 mm petri dish and grown overnight at 5% CO_2_ in a 37°C humidified incubator. Plasmid DNA (2 μg) was transfected into cells for 72 h using Effectene transfection reagent (Qiagen) according to the manufacturer's instructions. Following transfection, the cells were gown under selection pressure with 1 mg/ml Geneticin for 8 days until they formed colonies. The colonies were pooled together and cultured thereafter in DME-F12 1:1 supplemented with 10% fetal bovine serum and 1 mg/ml Geneticin.

### RNA silencing

Cells grown overnight in culture dishes were transfected with 50 nM scrambled control siRNA (Cell Signaling, Pickering, ON), 100 nM TRF2 siRNA and 100 nM RAP1 siRNA (both Santa Cruz, Santa Cruz, CA) for 48h using siLenFect lipid reagent (Bio-Rad, Mississauga, ON), after which nuclear protein extracts were collected for immunoprecipitation.

### Immunoprecipitation (IP)

The cytoplasmic fraction was first separated using the NE-PER kit according to the manufacturer's instructions (Thermo Scientific). The pellet was lysed using two volumes of the lysis buffer 50 mM Tris-HCl (pH 7.5), 150 mM sodium chloride, 25 mM sodium fluoride, 0.1 mM sodium orthovanadate, 0.2% Triton X-100, 0.3% NP-40, and protease inhibitors [69]. The mixture was incubated on ice for 40 min with intermittent vortexing every 10 min, followed by centrifugation at 20,800g for 30 min at 4°C. Supernatant containing the nuclear fraction was used for IP. The nuclear extracts were incubated with the primary antibody for 4 h at 4°C and then incubated at 4°C with a 1:1 ratio of protein G-agarose (Roche, Laval, Quebec) and protein-A sepharose (GE Healthcare, Baie-D’ Urfé, Quebec) beads for 16 h. The complex was washed thrice with the same lysis buffer and proteins were eluted by boiling the samples at 95°C in 3x SDS Laemmli buffer [70].

### Western blot

Protein extracts were separated by SDS PAGE, transferred onto a nitrocellulose membrane and incubated for 1 h at room temperature (RT) in blocking buffer specified by the antibody manufacturer. Primary antibody incubation was performed overnight at 4°C and secondary antibody incubation was for 1 h at RT. Primary antibodies used were TRF2 (mouse monoclonal, Novus Biologicals, Oakville, ON), RAP1 (rabbit polyclonal, Bethyl Laboratories, Montgomery, TX), POT1 (rabbit polyclonal, Abcam, Toronto, ON), HMGA2, α-tubulin, phospho-CHK2 (pCHK2^T68^), total CHK2 (rabbit polyclonal), Myc tag (71D10), phospho-CDC25C (pCDC25C^S216^), total CDC25C (5H9), phospho-ATM (D6H9) (pATM^S1981^), total ATM (D2E2) (rabbit monoclonal) (all from Cell Signaling), lamin A/C (N-18) (goat polyclonal, Santa Cruz), Flag tag (clone M2), β-actin (clone AC-15) (both mouse monoclonal, Sigma) and phospho-TRF2 (pTRF2^T188^) (kind gift from Dr. David Gilley, Indianapolis, IN). Secondary antibodies were horseradish peroxidase- (HRP-) conjugated goat anti-mouse IgG (Sigma), goat anti-rabbit IgG (Cell Signaling) and rabbit anti-goat IgG (Santa Cruz). For immunoprecipitated extracts, HRP-conjugated anti mouse IgG (Mouse True Blot, Rockland, Limerick, PA), anti-rabbit IgG from clean blot IP detection kit and chemiluminescent ECL substrate (both Thermo Scientific) were used.

### Immunofluorescence (IF)

Cells were seeded and cultured overnight on 3-aminopropyl triethoxysilane (APTES, Sigma) coated glass slides. The cells were fixed for 20 min in 3.7% formaldehyde (Fisher Scientific), permeabilized using 0.25% Triton X-100 and blocked with 4x SSC/4%BSA for 1 h at RT. Primary antibodies for HMGA2 (D1A7) (rabbit monoclonal, Cell Signaling) and TRF2 (mouse monoclonal, Novus Biologicals) were incubated for 1 h at RT followed by 1 h incubation at RT with secondary antibodies such as Alexa Fluor (AF) 488 conjugated goat anti rabbit IgG and AF594 conjugated goat anti mouse IgG (both Life Technologies). Slides were then counterstained with DAPI (Sigma), mounted with Vectashield (Vector Laboratories, Burlington, ON) and imaged with a Zeiss Z1 microscope using a 63x oil immersion objective with NA of 1.4 and Axio Vision Software (Zeiss, Jena, Germany). Following deconvolution, colocalization analysis was performed using the ImageJ colocalization plugin on single, segmented nuclei as described previously [71]. The colocalizing signals were extracted and displayed as a separate image. 50 nuclei were randomly analysed under each experimental condition and the average number of colocalizing spots per nucleus was graphed with error bars representing standard errors of the means.

### ImmunoFISH

C1 cells (untreated and dox treated) were grown overnight on APTES coated glass slides, fixed with 3.7% formaldehyde in PBS for 20 min at RT and permeabilized with 0.25% Triton X-100 for 10 min at RT. After blocking for 1 h at RT with 4% BSA in 4x SSC buffer, slides were incubated for 1 h at RT with primary antibodies to HMGA2 (D1A7) (rabbit monoclonal), 53BP1 (rabbit polyclonal, both Cell Signaling), TRF2 (mouse monoclonal). After washing, pepsin (Sigma) digestion was done for 4 min at 37°C. Slides were post-fixed in 3.7% formaldehyde for 2 min at RT followed by dehydration through ethanol series. Fluorochrome–coupled (Cy3) Telomere PNA probe (DAKO) was applied (5μl probe/slide), and following denaturation at 80°C for 3 min, hybridization was done for 2 h at 30°C. Slides were washed at RT in 70% deionized formamide (Sigma) in 10 mM Tris pH 7.4, followed by 2x SSC (5 min at 55°C), 0.1x SSC and 2x SSC/0.05%Tween-20 at RT. Secondary antibody AF488 goat anti rabbit IgG or AF488 goat anti mouse IgG (both Life Technologies) was applied to the slides and incubated for 1 h at RT. Slides were washed in 2x SSC/0.05% Tween 20, counterstained with DAPI (Sigma), mounted with Fluoromount G (Southern Biotech) and imaged on a Zeiss Axio Imager.Z1 using a 63x oil immersion objective with NA of 1.4. Axio Vision Software was used to capture Z-stack fluorescence images (45 per nucleus) at 0.2 μm intervals. Following deconvolution, a minimum of 50 nuclei from each treatment group were processed for colocalization using NIH Image J Software and Tools for Analysis of Nuclear Genome Organization (TANGO) software [72]. All structures were segmented using the stock segmentation; background was removed for FITC and Cy3 signals with a tophat filter. Signal quantification was performed for FITC (HMGA2, 53BP1 or TRF2) and Cy3 (telomere) signals and simple geometric measurements were taken for the nuclei and Cy3 signals. Finally, distance measurements between FITC and Cy3 signals were determined for colocalization purposes with any signals separated by a distance less than the optical resolution after deconvolution (102 nm) designated as colocalized.

### Quantitative telomere fluorescent *in situ* hybridization

Deconvolved images obtained from immunoFISH experiments were processed using TeloView [47, 51] and TANGO [72] software to determine the number of telomere signals, telomere aggregates per nucleus and the length of the telomeres.

### Proximity ligation assay (PLA)

PLA experiments were done using the Duolink kit (Sigma) according to manufacturer's instructions using the red detection reagents and Mouse Minus and Rabbit Plus reagents. C1 cells were grown on microscope slides with hydrophobic wells (CSM Inc. HTC supercured, white, 10 well, 7mm) coated with APTES (Sigma) and fixed for 30 min at RT in 3.7% formaldehyde. Following permeabilization in 0.25% Triton X-100 for 10 min, blocking solution (Duolink kit) was incubated on the cells for 30 min at 37°C. Primary antibody dilutions were made in the antibody diluent provided (Duolink Kit) and applied to respective wells for 1 h at RT. Antibodies used were HMGA2 (D1A7) (rabbit monoclonal), TRF2 (mouse monoclonal), RAP1 (rabbit polyclonal), rabbit IgG (DAKO), and mouse IgG (Sigma). After washing 2x in wash buffer A (Duolink kit), PLA probes were added according to manufacturer's instructions and incubated for 1 h at 37°C. This incubation was followed by 2x washing in wash buffer A. Ligation was carried out at 37°C for 30 min, then washed 2x in wash buffer A. Amplification was carried out in the dark according to manufacturer's instructions for 100 minutes at 37°C. Slides were then washed 2x in wash buffer B and 1x in 0.01x wash buffer B, then air-dried, coverslipped using the provided mounting medium with DAPI and imaged on a Zeiss Axio Imager. Z1 with Axio Vision software. Images were composed of 45 z-stacks at 0.2 μm thickness. Individual nuclei were deconvolved and a minimum of 30 nuclei was processed for PLA foci quantification using the Duolink Image tool software.

### Anaphase bridges and micronuclei

Cells were grown overnight on APTES coated glass slides and fixed with either methanol:acetic acid (3:1 ratio) or 3.7% formaldehyde. Following nuclear staining with DAP1, slides were coverslipped with Fluoromount G and imaged using Zeiss Z2 microscope and Zen Software. Slides were screened for anaphase bridge structures and micronuclei among a random 300 nuclei imaged. Triplicate experiments were performed and percentages of anaphase bridges and micronuclei were calculated.

### WST cytotoxicity assay

WST assay was performed according to the manufacturer's instructions to determine the EC_50_ for KML001 (Sigma). Briefly, 5000 cells per well were seeded in a 96-well plate format and cultured overnight. The cells were treated with increasing concentrations of KML001 for 24 h after which WST reagent (Roche) was added. Killing concentration was determined by measuring the absorbance at 450 nm using a 96-well plate reader (Perkin Elmer, Woodbridge, ON) 4 h after incubation with the WST reagent.

### Telomere - Chromatin immunoprecipitation (ChIP) assay

Ten million C1 (fibrosarcoma) cells per 150 mm dish (+/−dox treatment) were cultured overnight in a 37°C and 5% CO_2_ incubator. Two 150 mm dishes were used per experiment. The cells were washed once with PBS, cross-linked with 1% formaldehyde for 10 min and quenched with 125 mM glycine for 5 min at RT. The cross-linked cells were washed with 1x PBS, scraped and centrifuged at 1200 rpm for 3 min. The pellet was lysed with cell lysis buffer containing 5 mM PIPES (pH 8.0), 85 mM KCl and 0.5% NP-40 with protease and phosphatase inhibitors (Roche) for 25 min at 4°C with gentle rocking and then centrifuged at 2000 xg for 5 min. The pellet was then resuspended in MNase digestion buffer that constitutes 10 mM Tris-HCl (pH 7.5), 0.25 M sucrose and 75 mM NaCl with protease and phosphatase inhibitors and supplemented with 0.3% SDS. The mixture was incubated for 2 h at RT followed by 35 cycles of 5 sec on/30sec off sonication rounds. Sonication was performed on ice and the sonicated content was centrifuged at 17,000 xg for 10 min at 4°C. The supernatant was collected and resuspended in buffer containing 50 mM Tris-HCl (pH 7.5), 150 mM sodium chloride, 25 mM sodium fluoride, 0.1 mM sodium orthovanadate, 0.2% Triton X-100, 0.3% NP-40, protease and phosphatase inhibitors. The mixture was precleared using 60 μl of Protein A/G Plus agarose beads (Santa Cruz) per ml of lysate for 1 h at 4°C with rotation. The beads were then discarded upon centrifugation at 1000 xg for 3 min and the A_260_ of the lysate was measured. Equal amounts of A_260_ were aliquoted and used for ChIP. Some were saved for input. TRF2 antibody (mouse monoclonal, Abcam) was added at a ratio of 2:1 to the lysate (μg antibody: A_260_ of lysate) and incubated overnight at 4°C with rotation. Corresponding IgG controls were also employed. The mixture was further incubated for 3 h at 4°C with the addition of 10 μl of Dynabeads Protein G (Invitrogen) per A_260_ of the lysate. Following the incubation, the beads were washed once with 1 ml of four different buffers in the order as follows; Low Salt wash buffer (0.1% SDS, 1% Triton-X-100, 2 mM EDTA, 20 mM Tris (pH 8.1) and 150 mM NaCl), high salt wash buffer (0.1% SDS, 1% Triton-X-100, 2 mM EDTA, 20 mM Tris (pH 8.1) and 500 mM NaCl), LiCl wash buffer (250 mM LiCl, 1% NP-40, 1% deoxycholate, 1 mM EDTA and 10 mM Tris (pH 8.1)) and 1xTE buffer (10 mM Tris (pH 7.5) and 1 mM EDTA) for 5 min each at 4°C with rotation. Following the washes, the protein-antibody-chromatin complexes on the beads were eluted using 3x SDS Laemmli protein lysis buffer for Western blot detection of TRF2. To quantify the telomeric DNA bound to TRF2, the protein-antibody-chromatin complexes on the beads were eluted by incubation with 200 μl of elution buffer (1% SDS and 100 mM NaHCO3) for 30 min at RT. The elute (ChIP) and the input samples were reverse cross-linked upon overnight incubation at 65°C followed by treatments with 0.02 μg/ml RNase A for 30 minutes at 37°C and 0.5 μg/ml Proteinase K for 1 hour at 55°C. Final purification of the elute (ChIP) and input DNA were performed using the QIAquick PCR Purification Kit (Qiagen).

### Real time quantitative PCR (qPCR)

Purified genomic DNA from ChIP and input samples were examined for telomere sequences using StepOnePlus real time qPCR (Applied Biosystems, Life Technologies). A 20μl reaction mix was prepared with SYBR Select Master Mix at a final concentration of 1X (Applied Biosystems), forward and reverse telomere primers [73], PCR grade water and template DNA (ChIP and input samples). Each sample was prepared in three replicate wells in the plate. 40 cycles of qPCR was performed at 95°C for 15 s followed by 54°C for 2 min [73]. The telomere primers used are as follows;

Forward primer (tel 1): 5′-GGTTTTTGAGGGTGA GGGTGAGGGTGAGGGTGAGGGT-3′
Reverse primer (tel 2): 5′-TCCCGACTATCCCTA TCCCTATCCCTATCCCTATCCCTA-3′

ChIP (antibody) qPCR results were normalized to the data from the corresponding ChIP (IgG) controls. Fold enrichment of telomeres in the ChIP (antibody) samples relative to ChIP (IgG) was calculated in two steps. First the non-specific signals were adjusted by subtracting the mean Ct values of IgG from the mean Ct values of antibody (Ct_IP_ – Ct_IgG_). Next, the fold enrichment relative to the background IgG signal was calculated using the formula 2^−(Ct^_IP_^−Ct^_IgG_^)^ (https://www.thermofisher.com).

### Reverse transcriptase-PCR (RT-PCR)

The cDNA synthesis was performed using 1 μg of total RNA and random primer (Promega), at the following temperatures: 65°C for 5 min, 25°C for 10 min, 42°C for 50 min and 70°C for 15 min. The forward and reverse primers used to detect HMGA2 are as follows: F-huHMGA2: 5′-CACTTCAGCCCAGGGACAACC-3′; R-huHMGA2: 5′-CCTCTTCGGCAGACTCTTGTGA-3′. PCR conditions constituted an initial denaturation for 3 min at 95°C, followed by 40 cycles of denaturation at 95°C for 1 min, annealing at 63°C for 1 min and extension at 72°C for 2 min. The PCR was completed with a final extension step at 72°C for 10 min.

### Statistical analysis

Student's t-test and one-way and two-way analysis of variance were employed to determine statistical significance between the treatment groups. Bonferroni post-hoc statistical tests were performed for multiple comparisons. p-values for telomere signal intensities of C1 +dox vs. C1 −dox for both TANGO and TeloView were calculated using the two-sample Kolmogorov-Smirnov test. A p-value <0.05(*) was considered statistically significant.

## SUPPLEMENTARY FIGURES


