# Supplementary material for: High Mobility Group A2 protects cancer cells against telomere dysfunction
Source: Oncotarget. 2016 Jan 18;7(11):12761–82. doi: 10.18632/oncotarget.6938 (PMC4914320; doi:10.18632/oncotarget.6938)
Supplement: Supplementary file 1 [file oncotarget-07-12761-s001.pdf]

SUPPLEMENTARY FIGURES

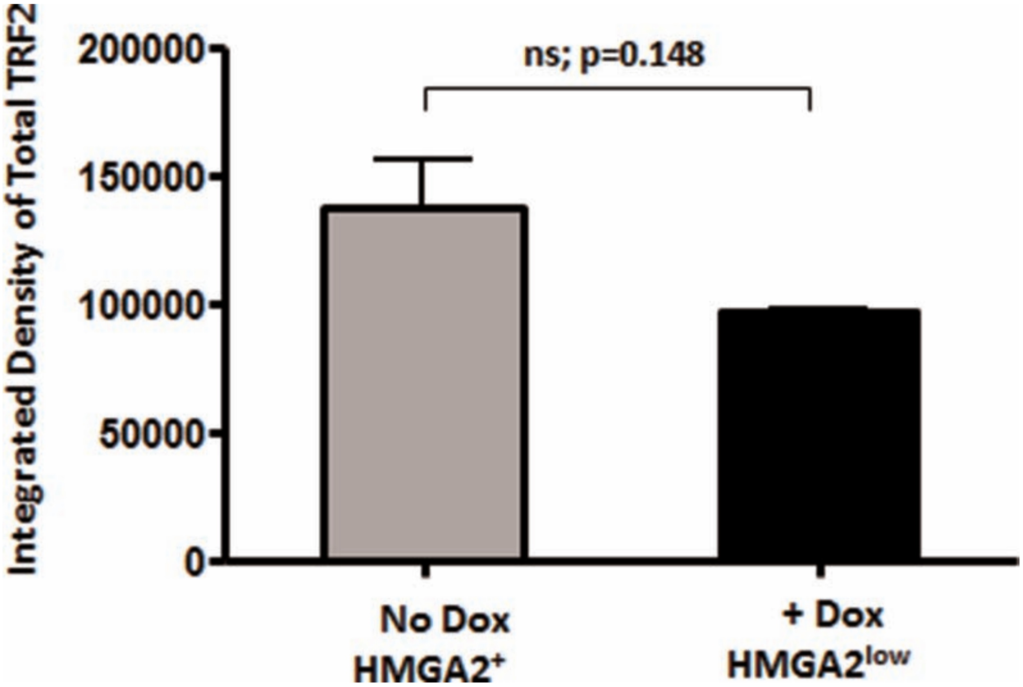

Supplementary Figure S1: Western blot was performed for semi-quantitative assessment of cellular TRF2 protein levels following shRNA-induced HMGA2 knockdown in the C1 cell model. Three independent experiments were performed and TRF2 protein was quantified from the integrated density. The differences were not statistically significant +/− HMGA2. Ns – no significant difference; p=0.148.

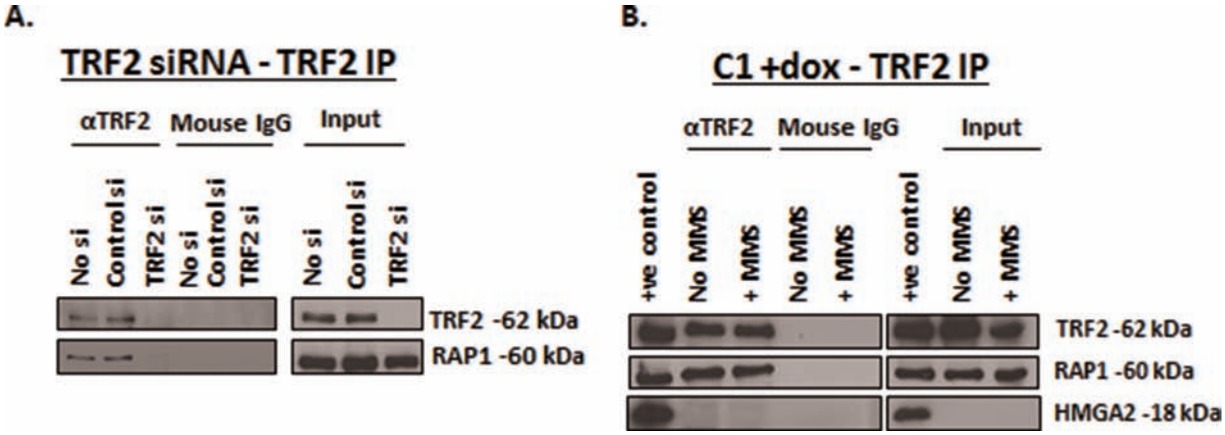

Supplementary Figure S2: IP was carried out from nuclear extracts of C1 cells to determine specificity of TRF2 and HMGA2 antibody used. A. TRF2 was knocked down by TRF2 siRNA and **B.** HMGA2 was knocked down by treatment with Doxycycline after which IP with anti-TRF2 was performed. **A.** In the TRF2 siRNA treated sample, both TRF2 and its known binding partner RAP1 were not detected upon pull down with the TRF2 antibody or with the isotype control antibody. **B.** HMGA2 was not detected in the dox-treated C1 sample ($HMGA2^{low}$) but RAP1 was still detected after TRF2 pull-down in HMGA2 silenced C1 cells showing the detection specificity of the antibodies used for IP and further confirming the effectiveness of the Dox treatment and the specificity of HMGA2 detection.

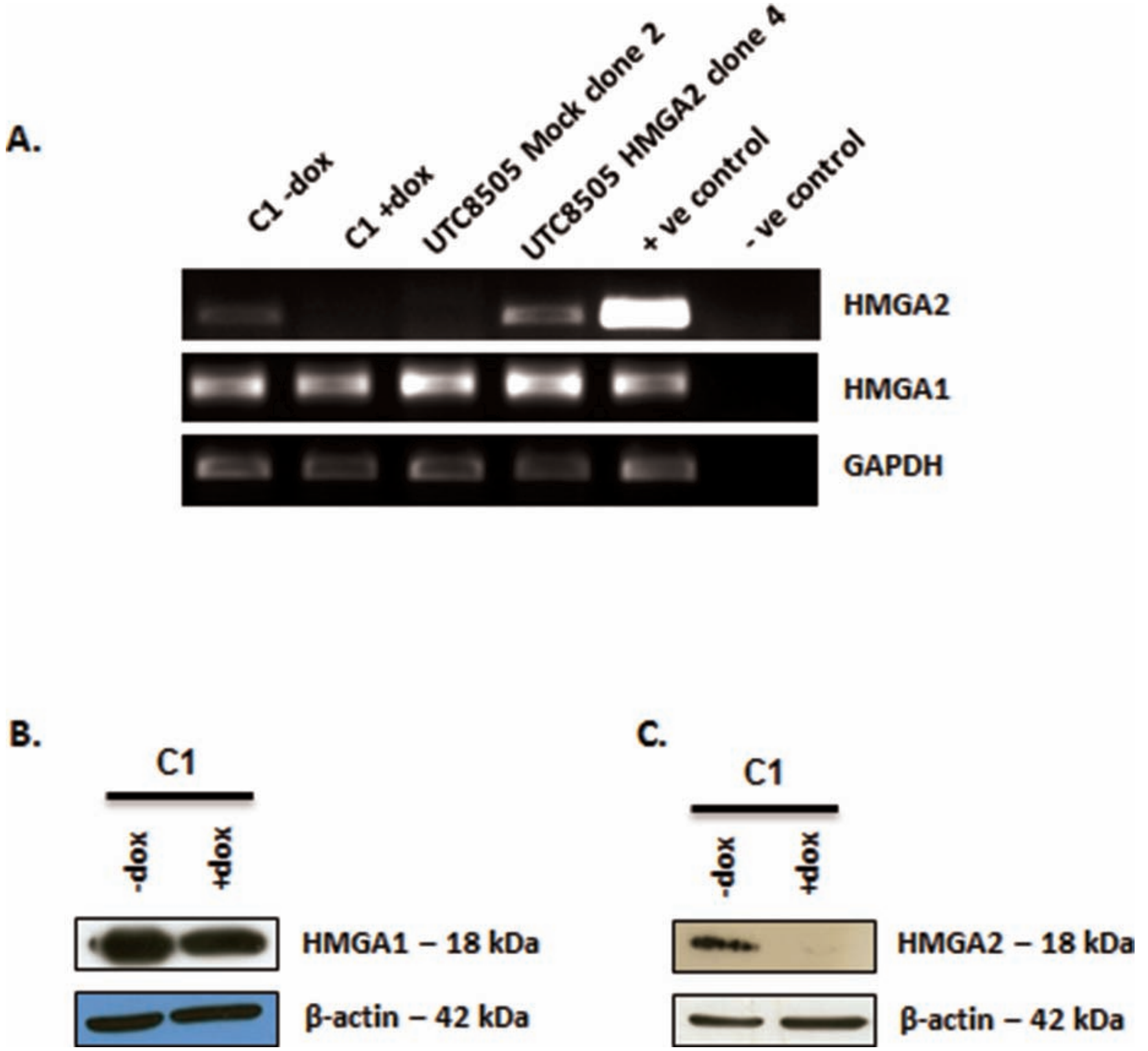

Supplementary Figure S3: A. Reverse Transcriptase PCR and **B, C.** Western Blot showing specific downregulation of HMGA2 but not HMGA1 upon doxycycline treatment in C1 cells. **A.** HMGA1 expression levels were also unaltered in the HMGA2 over-expressing transfectants of UTC8505 (Mock clone 2 and HMGA2 clone 4). GAPDH and β-actin served as loading controls.

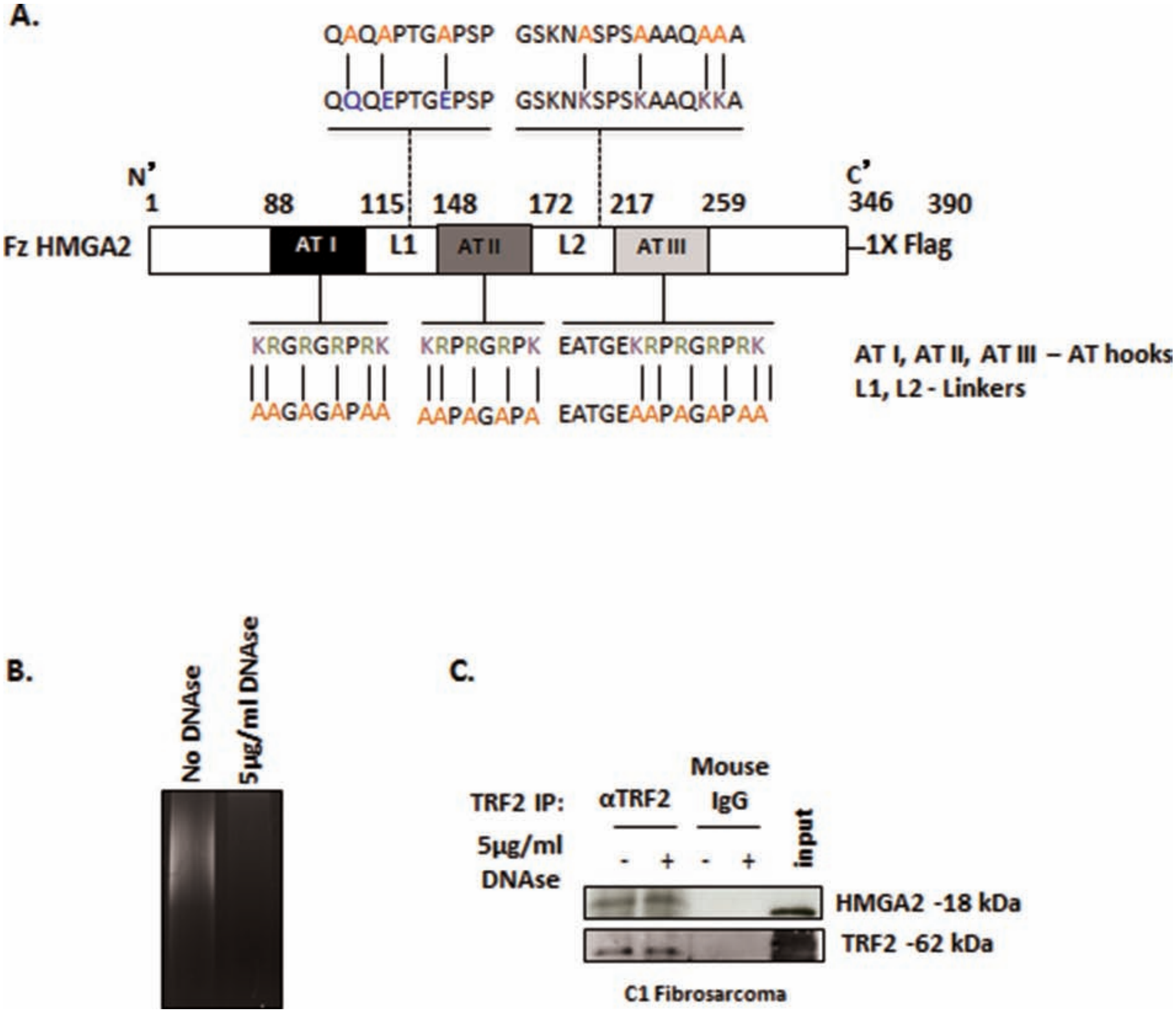

Supplementary Figure S4: A. Schematic depicting Flag-tagged HMGA2 AT-hook mutants and linker mutants with alanines [A] replacing arginines, lysines, glutamines and glutamic acid. **B.** Digestion of genomic DNA with DNAse. **C.** No inhibition of the HMGA2-TRF2 interaction was detected in the nuclear extracts used for IP upon DNAse digestion.

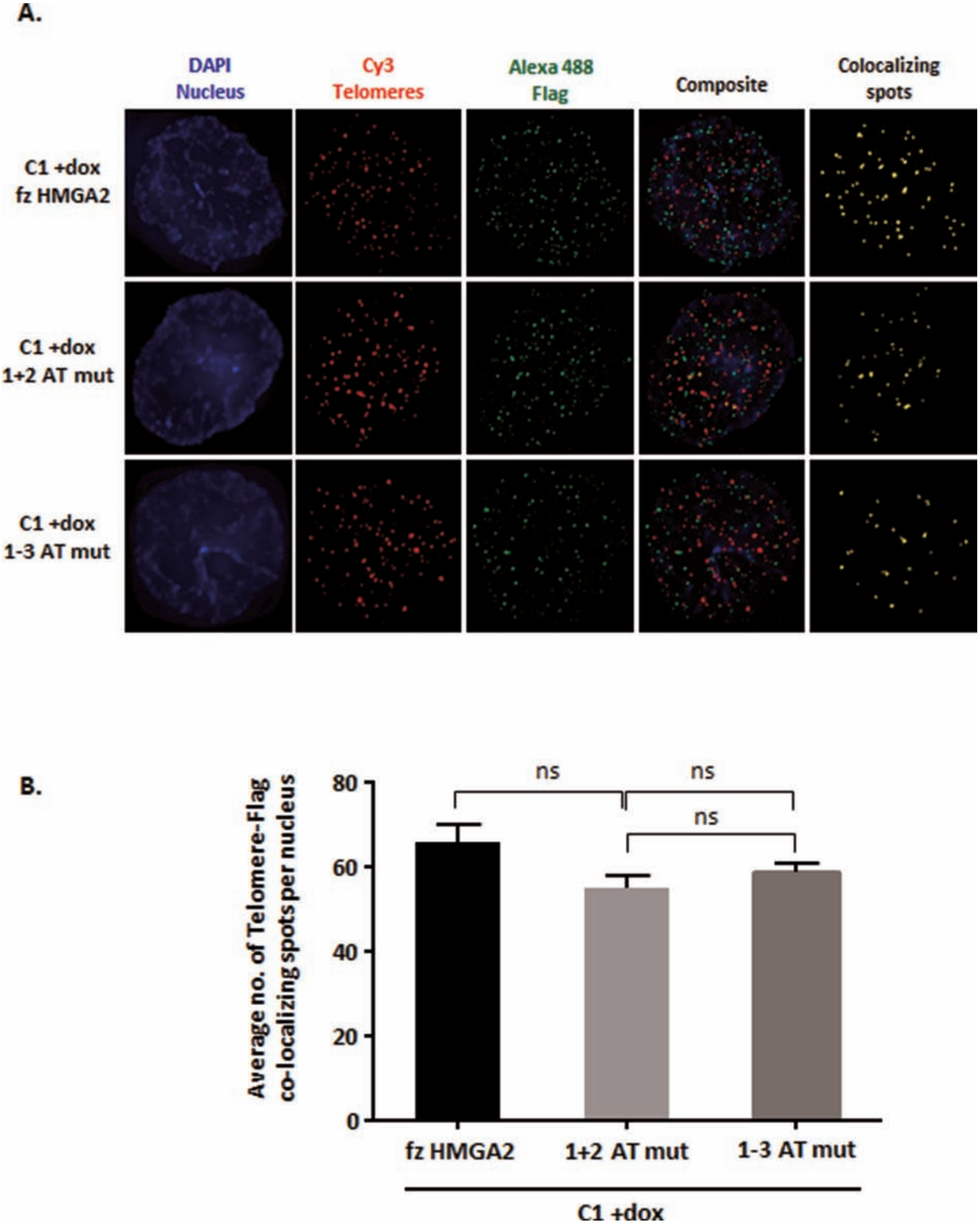

Supplementary Figure S5: A. Telomeric localization of Flag-tagged AT-hook mutants of HMGA2 by combined immunofluorescence (anti-Flag) and FISH (Cy3 telomere probe) analysis in +dox C1 cells transiently transfected with the AT-hook mutants Blue-Nucleus; Red-Telomeres; Green-Flag tag; yellow-Telomere-Flag co-localizing spots. **B.** Average number of Flag signals per nucleus co-localizing with telomeres were quantified and graphed. 50 nuclei were counted and the quantitative data are shown as mean +/− SEM; ns – no significant difference.

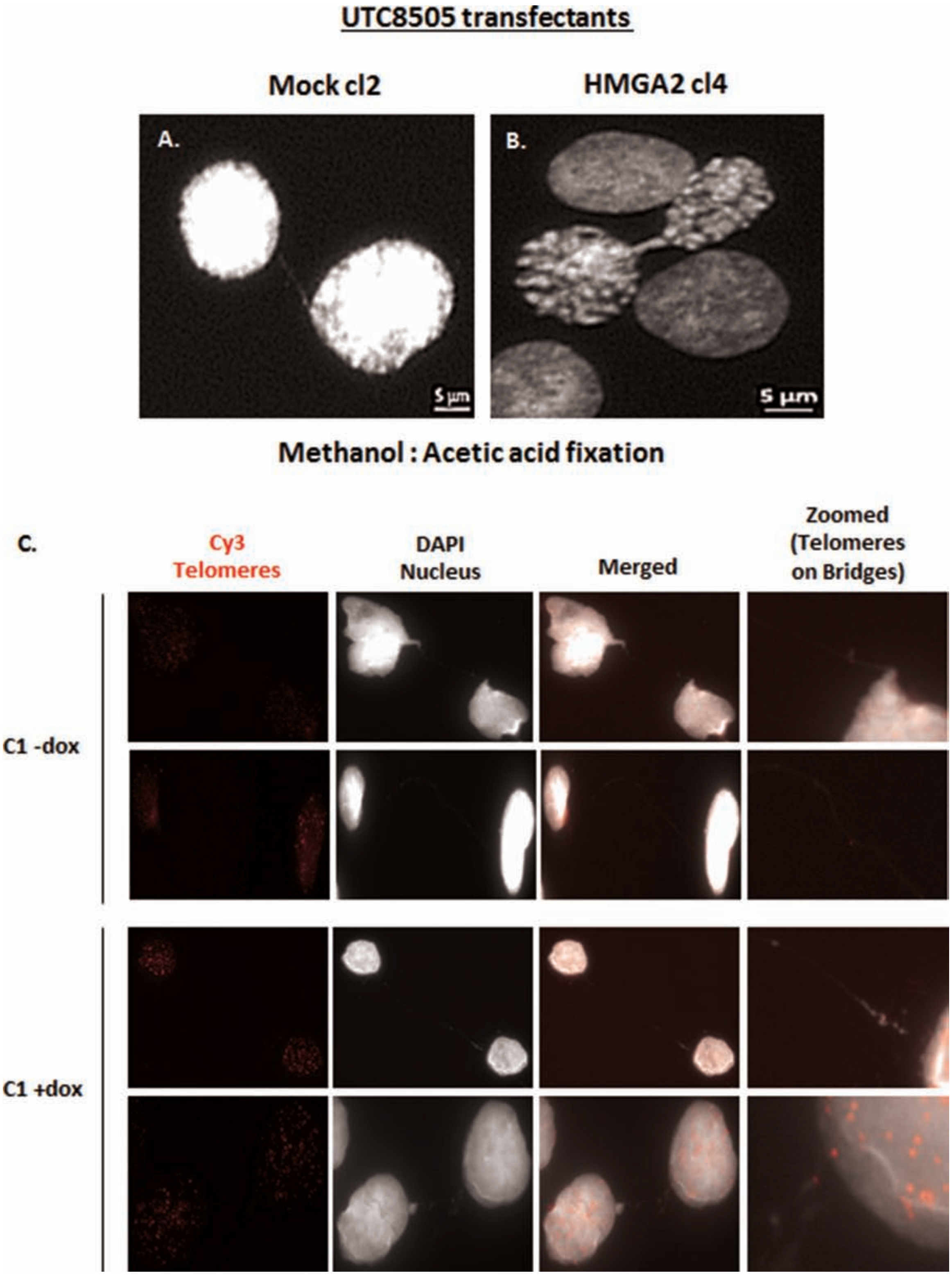

Supplementary Figure S6: Representative images of anaphase bridges in **A.** UTC8505 Mock clone 2 and **B.** UTC8505 HMGA2 clone 4 upon fixation with Methanol:Acetic acid. **C.** Telomere-FISH was performed in C1 cells +/−dox. Anaphase bridges contained telomere FISH signals in both HMGA2^{low} and HMGA2^{high} (+/−dox) C1 cells. Red-Telomeres; Blue-Nucleus (shown in black and white for better visualization of the Cy3 red telomere signal on the anaphase bridges).

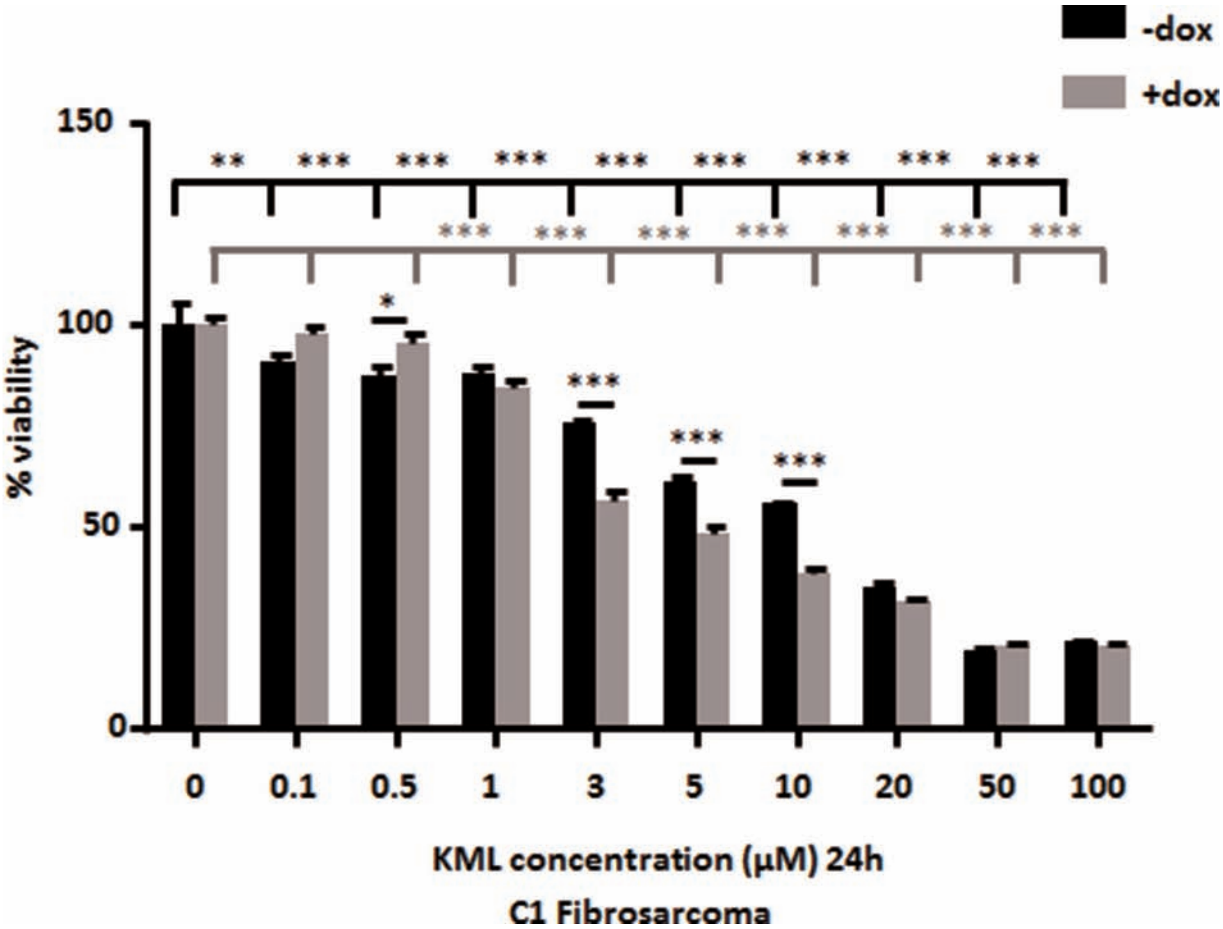

Supplementary Figure S7: WST assay was performed to determine cytotoxicity for different KML001 concentrations in C1 cells −/+dox treated for 24h. Significant differences in drug sensitivity were observed in the presence (-dox) and absence (+dox) of HMGA2. *p<0.05; **p<0.01; ***p<0.001.

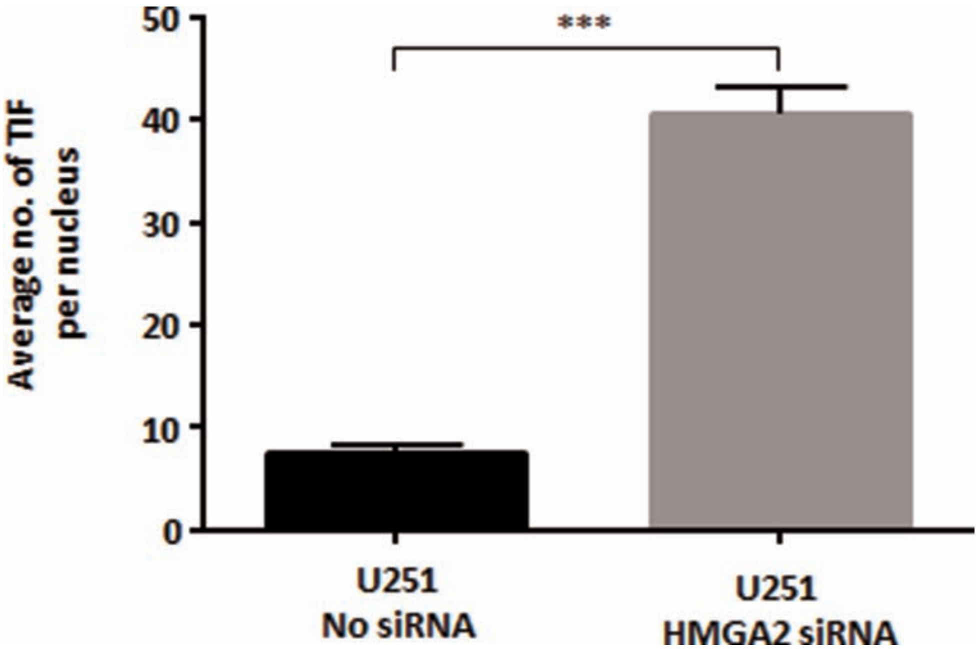

Supplementary Figure S8: Quantification of the average number of telomere-53BP1 co-localizing foci (TIF) per nucleus −/+ siHMGA2 in a glioblastoma cell model U251. Knockdown of HMGA2 in U251 cells resulted in a dramatic increase in TIF. Quantitative data are shown as mean +/− SEM; ***p<0.001.
